# MDMA-Assisted Therapy as a Means to Alter Affective, Cognitive, Behavioral, and Neurological Systems Underlying Social Dysfunction in Social Anxiety Disorder

**DOI:** 10.3389/fpsyt.2021.733893

**Published:** 2021-09-27

**Authors:** Jason Luoma, M. Kati Lear

**Affiliations:** Portland Psychotherapy Clinic, Research, and Training Center, Portland, OR, United States

**Keywords:** MDMA-assisted therapy, social anxiety disorder, social functioning, shame, social anhedonia, social threat

## Abstract

Social anxiety disorder (SAD) is a prevalent and often debilitating psychiatric disorder that can assume a chronic course even when treated. Despite the identification of evidence-based pharmacological and behavioral treatments for SAD, much room for improved outcomes exists and 3,4-methylenedioxymethamphetamine (MDMA) has been proposed as a promising adjunctive treatment to psychological interventions for disorders characterized by social dysfunction. A small randomized, placebo-controlled trial of MDMA-assisted therapy (MDMA-AT) for social anxiety in autistic adults offered encouraging results, but more research is sorely needed to explore the potential for MDMA-AT in treating SAD. This review aims to stimulate future study by summarizing research on disruptions in neurological, perceptual, receptive, and expressive systems regulating social behavior in SAD and proposing how MDMA-AT may alter these systems across four domains. First, we review research highlighting the roles of social anhedonia and reduced social reward sensitivity in maintaining SAD, with specific attention to the reduction in positive affect in social situations, infrequent social approach behaviors, and related social skills deficits. We posit that MDMA-AT may enhance motivation to connect with others and alter perceptions of social reward for an extended period following administration, thereby potentiating extinction processes, and increasing the reinforcement value of social interactions. Second, we review evidence for the central role of heightened social evaluative threat perception in the development and maintenance of SAD and consider how MDMA-AT may enhance experiences of affiliation and safety when interacting with others. Third, we consider the influence of shame and the rigid application of shame regulation strategies as important intrapersonal processes maintaining SAD and propose the generation of self-transcendent emotions during MDMA sessions as a mechanism of shame reduction that may result in corrective emotional experiences and boost memory reconsolidation. Finally, we review research on the role of dysfunctional interpersonal behaviors in SAD that interfere with social functioning and, in particular, the development and maintenance of close and secure relationships. We discuss the hypothesized role of MDMA-AT in improving social skills to elicit positive interpersonal responses from others, creating a greater sense of belonging, acceptance, and social efficacy.

## Introduction

Social anxiety disorder (SAD) is the fourth most commonly diagnosed psychiatric disorder in the United States (1). Approximately 13% of the population in the U.S. will have SAD at some time in their life, with as many as 8% reporting SAD over the past year (1). SAD is characterized by a frequent and intense fear in social situations in which the person is afraid of being scrutinized by others and, as a result, avoids or experiences social situations with significant tension or discomfort (2). Epidemiological research has found that people with SAD report overall reduced quality of life, with negative consequences on their social and occupational lives, even at levels below the diagnostic threshold (3, 4). Indeed, behaviors associated with SAD have been shown to contribute to delayed and fractured relationships, reduced likelihood of partnering and having children, workplace absenteeism, lower worker productivity, and inability to maintain or initiate employment (2, 5, 6). SAD is often associated with the concurrence and development of other psychological disorders, particularly alcohol use disorder (7, 8) and major depressive disorder (MDD) (9). Although the exact nature of these relations remains unclear, SAD often occurs first and confers approximately a four-fold risk and five-fold risk for developing alcohol dependence (7) and MDD (10), respectively, relative to the general population. SAD may also confer greater risk for adverse physical health outcomes. SAD increases the risk for chronic social isolation and loneliness, robust longitudinal predictors for cardiovascular disease and increased mortality (11).

Among people with SAD, intense fears of being negatively evaluated in social situations often arise during adolescence (12) and the problem often goes unrecognized as something that could be treated (13). Evidence-based pharmacological (14) and behavioral treatments (15) for SAD have been identified, but much room for improved outcomes exists. Meta-analytic data on medication-based therapies suggests that SSRIs and SNRIs are associated favorable outcomes compared to placebo yet are also associated with higher dropout rates over the treatment course (14, 15). The efficacy of pharmacotherapies also appears dependent on continued adherence to medication, with as many as 25% of people reporting relapse after discontinuation of the drug. In terms of psychotherapies, cognitive-behavioral therapy has shown strong evidence for treatment efficacy and superior longevity to pharmacotherapy (15). Yet, in the case of both medication and psychotherapy a significant proportion of patients remain considerably symptomatic at the end of treatment (15), with social anxiety often assuming a chronic course in spite of intervention (16).

Recent research has begun to explore whether certain drugs can be used to augment the effectiveness of psychological interventions for SAD. A recent systematic review of d-cycloserine as a means to augment psychotherapy for SAD found that it provides a small augmentation effect for exposure therapy in treating SAD (17). A second drug that has been investigated as an augmentation strategy in the treatment of SAD is 3,4-methylenedioxymethamphetamine (MDMA). A small randomized, placebo-controlled trial of MDMA-assisted therapy (MDMA-AT) for social anxiety in autistic adults (*N* = 12) found a large between groups difference in social anxiety symptoms at the primary endpoint in favor of MDMA-assisted therapy (*n* = 8; seven completed treatment) vs. psychotherapy plus placebo (*n* = 4*; d* = 1.4) after two doses of MDMA. Results from this trial suggest promise for the treatment of SAD in the absence of autism. Although autism spectrum disorder appears to confer greater risk of developing SAD relative to the general population, clinically elevated levels of social anxiety are found only in the minority of individuals with autism, demonstrating that SAD is a problem separable from autism itself, while still sharing some features (18). MDMA-AT has also been most extensively tested in the treatment of post-traumatic stress disorder (PTSD) and has shown to result in large effect size differences compared to placebo controls in Phase II and Phase 3 trials (18–21). In addition, both phase II (19) and the existing phase 3 study (20) suggest excellent safety and low abuse potential when MDMA is used under controlled conditions. However, little research has been conducted on its possible mechanisms of action in the context of treatment studies.

MDMA has been proposed as a particularly promising treatment adjunct for disorders characterized by social dysfunction, such as SAD (22). This may be due, at least in part, to its stimulation of hormonal responses related to oxytocin, vasopressin, prolactin, and cortisol (23–25), all of which have profound impacts on social behavior. These effects are largely downstream of primary effects of MDMA on serotonin, norepinephrine, and dopamine systems (26). MDMA also functions as a stimulant and results in subjective effects including, most relevant to this paper, altered responses to social stimuli and experience of social emotion, including heightened feelings of empathy, and feelings of love (27) and compassion (28).

MDMA-assisted therapy is typically delivered in the following format. Treatment begins with three preparatory sessions aimed at helping participants get ready for dosing sessions by providing information, answering questions, exploring relevant history, and teaching preliminary skills. This is followed by a dosing session, which is then followed by integration sessions aimed at helping the person make sense of what happened in the dosing sessions and how to generalize what they learned into their daily life. Studies typically have a total of 2-3 dosing sessions, each followed by three integration sessions.

Alongside the promise of MDMA-AT in augmenting current therapies for SAD to ameliorate social anxiety symptoms, evidence remains preliminary. One factor that may limit progress in this area is the lack of clear theorizing on potential processes of change that may occur in the context of MDMA-AT. A stronger theory will help identify potential processes of change that could guide research to improve integration between drugs and psychotherapy.

To address this gap in the literature, this review summarizes data on alterations in the affective, cognitive, and neurological systems related to social functioning in SAD and how MDMA-assisted therapy may alter these functions. We focus not on the immediate effects in dosing sessions, which we discuss in another paper (Luoma et al., manuscript submitted), but rather on downstream effects that may occur in the weeks or months after dosing sessions.

We discuss four overarching domains in which therapeutic processes of change may occur and result in long-term improvements in functioning in SAD. First, the potential role of MDMA-AT in reducing social anhedonia and enhancing social reward sensitivity in SAD. Second, the role of social threat perception and functioning of the ventral vagus complex and associated neurological structures in SAD and how MDMA-AT may result in sustained alterations in their functioning. Third, the role of shame and shame-regulation strategies, such as self-criticism, in maintaining elevated threat perception in SAD and how MDMA-AT may interrupt these patterns. Fourth, the potential role of MDMA-AT in changing interpersonal behavioral patterns observed in SAD that may maintain the disorder.

We present these domains as distinct for the purpose of presentation clarity; however, we view them as related and interdependent processes of change that reciprocally affect one another to affect overall social functioning in SAD. This conceptualization has guided terminology used throughout this paper in that we have chosen to use the term process of change, rather than mechanism, as it allows for the existence of bidirectionality, feedback loops, and concurrent change, whereas mechanism often implies a linear or causal pattern of variable interaction (29).

## Social Anhedonia And Social Reward Sensitivity

People with SAD often have broad impairments in positive emotions, particularly in response to social situations (30, 31). For example, people with SAD experience diminished positive affect and increased negative affect in social interactions in general (32), and reduced positivity in response to social acceptance and enhanced negativity in response to social rejection (33). The reduction in positive affect in response to social situations, infrequent social approach behaviors, and related social skill deficits observed in SAD has been termed social anhedonia (31). Relatedly, people with SAD show altered responses to social reinforcement, leading to alterations in the balance between approach goals related to seeking social connection and advancing socially and avoidance goals related to social threat (34, 35). An experimental study showed that due to greater hypersensitivity to social punishment, people with social anxiety may have greater difficulty adapting to increased social reinforcement after being rejected by someone (or perceiving they were) even when that person later becomes more rewarding (36). This overactivation of avoidance goals may be part of why people with SAD tend to interpret positive social stimuli in threatening ways (30) and show increased fear of positive evaluation from others (37). For example, people with SAD often react to positive feedback by expecting that their partner will expect more from them in the next interaction and that they will fall short of those expectations (38, 39). This tendency to interpret positive social events as threatening has been shown to partially mediate the relationship between social anxiety and decreased positive affect (40). Increasing the relative balance of approach vs. avoidance goals as it relates to social situations may be core to recovery from SAD. For example, increased approach behavior in the form of sharing positive emotions is associated with improvements in SAD symptoms during treatment (41) and experimental reductions in safety behaviors (i.e., reduced avoidance) among people with high social anxiety (SA) results in more enjoyment during social events (42, 43). Together, these results suggest that increased positive reinforcement in social situations and reduced experiences or perceptions of punishment are important in the treatment of SAD. In relation to MDMA-AT, various sources of evidence suggest that MDMA may make social interactions more rewarding and less punishing and that these effects may endure beyond the dosing session. This may help alter the ratio of approach- vs. avoidance-focused social goals that appear to maintain SAD. MDMA has been shown across multiple studies to affect the action of brain systems related to social bonding and positive reinforcement related to social encounters (22). At a subjective level, people ingesting MDMA report feeling peace, safety, and love (27) and a subjective sense of desire to be with others (44), which comports with observations that MDMA increases motivation to affiliate with others during active dosing. At a neurochemical level, MDMA has been shown to induce the release of oxytocin and prolactin, two hormones centrally implicated in the ability to form social connections (45, 46). MDMA also strongly affects 5-HT2A receptors, which are important mediators of behavioral sensitization (47–49). A recent series of studies on mice indicate that the effects of MDMA on social behavior may extend beyond acute dosing. In a series of experimental studies, Nardou et al. (50) showed that mice exhibit a critical period through adolescence when they find social encounters with stranger mice reinforcing but then lose this preference as they age. Nardou et al. (50) also demonstrated that this critical window can be reopened in adult mice by administering MDMA in the presence of another mouse. Furthermore, this increased sensitivity to social reinforcement extended for at least 2 weeks post-administration. They also revealed that this effect did not occur with cocaine administration, suggesting it was not simply the pairing of positive stimuli with the presence of another mouse that resulted in the effect. Additionally, they also established that the MDMA needed to be administered with another mouse present in order to achieve this enduring shift in social reinforcement, suggesting that the social context of MDMA administration is important. Another series of mouse studies by Curry et al. (51) showed increased social behavior toward unfamiliar mice after multiple treatments with MDMA, with this effect being greater when the mice received MDMA while in the presence of another mouse. Together, these results suggest that MDMA may enhance motivation to connect with others, enhance the likelihood that people find social interactions with strangers rewarding, and that this effect may extend beyond MDMA administration in a therapeutic, social context. These effects could have multiple therapeutic benefits in people with SAD. First, it could shift motivation to engage in social behavior that could potentiate extinction processes in post-dosing sessions. For instance, the only available placebo-controlled trial testing MDMA-AT on social anxiety symptoms in autistic adults (52) found that, out of the seven participants who completed a full course of MDMA-AT, two people reported initiating dating for the first time, and another two reported increased comfort exploring and expressing gender identity at the 6-month follow up. It is possible that temporary increases in the reinforcement value of social interactions could initiate a new pattern of behavior in which individuals with SAD seek out previously avoided social situations and have more frequent positive social experiences, resulting in a positive chain of events in which affiliative behavior toward others elicits reciprocal affiliative behavior toward oneself [e.g., (53)]. Over time, this new learning could reinforce social approach and result in a reduced perceived need to enact safety behaviors that interfere with effective social functioning thereby increasing perceptions of social efficacy.

## Heightened Social Threat

A heightened tendency to experience social stimuli as threatening has been posited to be *the* central mechanism in the development and maintenance of SAD (12). People diagnosed with SAD show heightened amygdala activity to stimuli related to social-evaluative threat, such as negative emotion expressions or critical comments about oneself (54–56). Essentially, people with SAD do not feel safe in their relationships with others. This appears to be particularly true when interactions involve greater threat of revealing their authentic self, such as in laboratory tasks focusing on closeness generation through reciprocal self-disclosure (57). In fact, those with higher social anxiety have been shown to self-disclose less often in social tasks when they have high expectations of being liked by another person, whereas this pattern is inverse for people with low social anxiety (58). Other examples of this pervasive sense of threat in interpersonal relationships is the greater discomfort with social touch among people higher in social anxiety (59), heightened levels of interpersonal distrust (60), increased amygdala activity in response to emotional stimuli (61), and heightened loneliness in SAD youth (62). This heightened interpersonal-evaluative threat may account for at least some of the impairments in interpersonal functioning seen in SAD. The ability to feel safe around others and respond to social interactions in flexible, fluid, and contingent ways appears to be mediated through the same neuroanatomical systems. This system has been termed the social engagement (63) or social safety system (64) and involves the functioning of the parasympathetic nervous system and most centrally the vagus nerve and ventral vagus complex. The branch of the vagus nerve most fundamental in regulating social engagement enervates the heart as part of regulating global metabolism, slowing the body, and fostering a state of quiescence and potential for social engagement. The activation of this ventral branch of vagus nerve is thought to be indexed by higher heart rate variability, which refers to regular fluctuations in heart rate linked to the breathing cycle (65). Reduced high frequency heart rate variability at rest, which may be measured in a variety of ways we will not outline here, has been linked to a variety of anxiety disorders, social anxiety in particular (66), and also to perceived social connection (45, 46). High frequency heart rate variability is thought to measure the operation of the vagal brake, which refers to the action of the parasympathetic nervous system in downregulating physiological arousal in situations that are perceived as safe and in which rest is an adaptive response. Research suggests chronic under activation of the vagal brake in individuals with SAD, although it is unclear whether vagal nerve impairments play a role in the development of SAD or are better conceptualized as SAD sequelae. Nevertheless, people with social anxiety disorder demonstrate a rigid response style to social situations that involves the inability to inhibit threat-based responses in non-threatening situations (66) and which can be seen in decreased heart rate variability at rest compared to healthy controls (67). In addition to allowing the body to rest and regenerate, the ventral vagus and associated central nervous structures are also responsible for enervating muscles associated with flexible and responsive social engagement in the face, neck, and larynx. The result is that when the ventral vagus is active, our voice is more musical and face is more mobile thus facilitating better social engagement (64). Thus, in situations of increased sympathetic activity and decreased parasympathetic activity, peoples' ability to respond to social situations in a sensitive and synchronous manner is impaired. In sum, the tendency for people with SAD to experience heightened social threat appears to result in decreased operation of the social engagement system during social events, resulting in dysfunction in the operation of the vagus nerve, and thereby difficulties in non-verbal flexible social interaction. Behavioral evidence of social miscoordination can be seen in evidence that people with SAD demonstrate impairment in the non-verbal synchrony in social situations that is associated with greater warmth and rapport (68). Furthermore, people with SAD do not show increases in non-verbal synchrony that occur in healthy controls when engaging in closeness-generating conversations (69). Further indicators of social miscoordination in SAD include reduced reciprocal giving (70) and greater sensitivity to social punishment that may result in a greater difficulty adapting to increased social reinforcement after being rejected by someone (36). People with SAD also show differential cortisol reactivity in a high self-disclosure context and evidence less of a decline in cortisol when self-disclosing compared to people lower in social anxiety (71). Different types of evidence indicate that when people with SAD attend to positive affiliative signals, this leads to a range of positive outcomes (72–74), suggesting that shifts in felt social safety, along with attendant shifts in the functioning of the social engagement system could mediate changes in social anxiety symptoms. In addition, several longitudinal studies combine in suggesting that close positive relationships and experiences of social mastery/belonging serve as protective factors against SA (30). For example, a prospective study of SA among adolescents found that SA decreases when positive friendship qualities (i.e., companionship and intimacy) increase (75). Other examples of the importance of attending to positive social stimuli can be found in research showing that people with SAD differed from healthy controls only in positive affective empathy, but not negative affective empathy (76). In other words, they could share in others' negative emotions in a laboratory task but were less able to vicariously share others' positive emotions. Improvement on an empathy accuracy task mediated treatment outcomes in group CBT for social anxiety; this was particularly the case for positive affective empathy relative to negative affective empathy (41). In addition, people high in social anxiety appear to underestimate intensity and duration of positive affect when exposed to a positive interpersonal vignette as compared to individuals low in social anxiety, and overestimate other-related disgust, internal shame, and guilt (77). This literature suggests that improvements in SAD are likely to be dependent upon the extent to which people experience a greater sense of safety in social encounters and that this change will be mediated through the social engagement system. MDMA-AT appears to help people feel safer with others. Anecdotal reports of early therapists using MDMA indicated that clients often report greater ease in relating in both close and more distant relationships for weeks or months afterwards (28). A qualitative analysis of reports from a clinical trial of MDMA-AT for PTSD found that reports of feeling safe were common, such as in the following quotes from participants,

… I think it allowed me to feel safe in my space… Now it was safe and I had my tools and weapons to be able to tackle the obstacles that I never had before; There's no words that's going to explain this but I have never in my life relaxed like I did during this…Maybe like what you would feel in your mommy's tummy or something, whatever being back in the womb, very safe, very warm [(78), p. 46].

Another possibility is that MDMA may potentiate changes in social safety through facilitating a greater focus on cues related to affiliation and intimacy and less of a focus on cues related to hierarchy and social rank. In most social situations, people with SAD may tend to focus on managing and attempting to improve a self-perceived low social rank, rather than on affiliation and intimacy (35). A focus on social rank, particularly in the context of perceived low social rank, is likely to activate emotion systems linked to defensive arousal and deactivate those related to social engagement (79). This tendency to perceive affiliative cues as threatening can be seen in the fear and avoidance that people with SAD show to the positive evaluations of others (80) as well as their tendency to respond to positive evaluations with elevated arousal compared to healthy controls (81). MDMA has been shown to facilitate responding to positive social cues preferentially (82). Thus, MDMA may bolster internally experienced positive affect by shifting clients' focus toward positive aspects of social experiences (e.g., being accepted and feeling connected), exploring positively-valenced emotional experiences, and formulating social approach goals (relative to avoidance). As reviewed above, this effect may have its action through the influence of MDMA on the bonding-related hormones such as oxytocin and prolactin that may be linked to vagal tone (83). In addition, the serotonin system which MDMA strongly affects has been implicated in bonding. For example, experimental research on prairie voles shows that traumatic events reduce pair bonding in prairie voles, but this is reversed by selective serotonin reuptake inhibitors (84).

## Shame And Shame-Related Coping

Different types of evidence point to the significance of shame in maintaining heightened social threat perception in SAD. Shame is a self-conscious emotion characterized by the perception of being inferior, unlikable, or unacceptable and a desire to hide the undesirable self from others (85). Negative self-representations, negative interpretation biases, negative self-imagery, and social-evaluative thoughts characteristic of shame are observed at higher levels among individuals with SAD than healthy controls [see (12) for a review]. Indeed, shame is more highly correlated with SAD than other anxiety disorders (86) and predictive of social anxiety symptom severity (87). Decreases in shame-proneness, but not guilt-proneness, are associated with decreases in SAD symptoms (88). Shame is theorized to have evolved to prevent humans from behaving in ways that have a high negative social cost and could result in social devaluation by others and loss of social rank (e.g., cheating, stealing) (89, 90). When people are informed that social devaluation has occurred, or perceive that it could occur, shame is triggered to organize their behavior around mitigating their loss of social standing and reducing risk of ostracism (91). Studies have shown that self-reported shame elicited by social vignettes is highly correlated with actual ratings of social devaluation among individuals within and across distinct cultures (90). Experimental research has also shown that shame is elicited by predictive cues of social devaluation, such as being told one would be excluded by others, regardless of whether they acted in a morally transgressive way, suggesting the experience of shame is intimately tied to one's sense of threatened social cohesion rather than specific social behaviors (91). For individuals with SAD, negative attentional biases associated with increased social threat perception may result in an overestimation of the potential for social devaluation across social contexts (12), resulting in more frequent and intense experiences of shame compared to people without SAD (92). Some clinical models of SAD treatment hold that the anxiety experienced by people with SAD is a secondary response to the anticipation of exposing the core shameful self to others (93, 94). Preliminary support for this idea is suggested by intensive longitudinal and experimental data. For example, a daily diary study of college students showed that social anxiety symptom severity predicted higher shame during significant social interactions among college students (95). Similarly, the likelihood of shame after receiving negative feedback following a speech task is more strongly predicted by trait social anxiety rather than ratings of state social anxiety immediately following that task (96). In this view, SAD is maintained because individuals with SAD develop a range of shame avoidance strategies that function to protect them from experiencing rejection, ostracism, and shame that may confirm predictions based on shame-based self-referential thinking (e.g., “I'll make a fool of myself”). These avoidance behaviors, however, inadvertently prevent them from working through their core sense of shame and reinforce anxiety in social situations. Self-criticism is a key internal shame-avoidance strategy in SAD (95, 97). In moderate doses, self-criticism can serve an adaptive self-monitoring function that serves to identify discrepancies between an individual's behavior and societal expectations, helping the person appropriately modify their actions to adhere to social norms. Among individuals with SAD, however, self-criticism appears to be applied frequently and inflexibly around social interactions (97) and incorporates a high level of negative self-evaluation (96, 98). For example, in a daily diary study, college students wither higher social anxiety reported increased self-criticism following social events, regardless of the level of shame they experienced, whereas those low in social anxiety only endorsed self-criticism following high-shame interactions (95). This inflexible pattern of self-criticism is thought to perpetuate anxiety in social situations by reinforcing beliefs of low social rank and inferiority (79) and triggering defensive arousal associated with a sense of social threat. Indeed, an fMRI study showed that self-criticism was associated with increased activation in the lateral prefrontal cortex and dorsal anterior cingulate, areas of the brain associated with error processing and behavioral inhibition, but not areas associated with mindfulness and self-reassurance, such as the insula, suggesting a heightened attentional focus on identifying and correcting mistakes rather than general self-reflection (99). Over time, negative attentional biases in SAD may manifest in a general negative self-concept (100), greater access to negative self-related imagery in social situations (101) and heightened access to negative autobiographical memories (102), serving to maintain the shame-bound sense of self among individuals with SAD. Reductions in shame would have a broad range of positive effects for individuals with SAD. For instance, general decreases in shame should reduce the accessibility of negative self-imagery (103) and the tendency to engage in self-critical rumination in response to stressful social events (95, 96, 104), both of which appear to serve as maintaining factors in SAD. More broadly, if revealing the authentic self is less likely to trigger shame, then defensive self-focused attention may also be reduced (105–107), resulting in a decreased need to regulate shame through avoidant, self-critical processes. Indeed, a study of emotion-focused therapy for social anxiety showed that decreases in shame expressed in-session were associated with reduced self-criticism the following week (92). MDMA-AT may help individuals with SAD reduce shame by facilitating experiences of compassion or kindness toward the self through neurochemical changes, as well as through modeling from the therapist in the dosing session. With respect to the former, two experimental studies with ecstasy (presumably containing MDMA in one study and tested to confirm MDMA in the other) demonstrated that ecstasy increases feelings of self-compassion and reduces self-criticism (108, 109). In addition, anecdotal reports of MDMA-AT sessions suggest that MDMA increases self-compassion and self-acceptance (28, 110). Research on SAD suggests that greater self-compassion is associated with decreased fear of positive and negative evaluation (111) and reduced social anxiety severity (112). In addition, MDMA appears to elicit self-transcendent emotions more broadly. The mostly commonly reported experience while taking MDMA is a blissful state characterized by feelings of pleasure, peace, and love (27). Love and compassion are self-transcendent emotions, a family of emotions that increase prosocial behavior (113, 114), perceptions of connectedness (115), and the tendency to transcend self-centered needs to focus on the needs of others (116). Self-transcendent emotions generated through loving kindness meditation have also been shown to increase social connectedness (117, 118) and reduce self-criticism (119). MDMA-AT also appears likely to strengthen the bond between therapist and client and boost feelings of safety in dosing sessions, potentially laying the foundation for reproducing these types of safe interactions with others following participation in MDMA-AT. MDMA-AT participants often report feeling very safe with therapists during dosing sessions (78) and the tendency for MDMA to elicit expressions of warm and positive emotions toward therapists is also likely to elicit reciprocal responding from therapists (120, 121). Furthermore, the tendency for MDMA to strengthen emotional responses to positive social stimuli (44, 122) is likely to make therapists' responses of compassion and warmth more salient at a visceral level. If the core fear of people with SAD is that the exposure of a seemingly flawed and inferior self will result in rejection and shaming from others (94), then this enhanced therapeutic bond should potentiate corrective interpersonal learning experiences by both encouraging the person to be authentic and thereby reveal this core defective self, while also resulting in a prediction error when the person experiences acceptance and caring as a response, rather than the expected ridicule or rejection. This could enhance memory reconsolidation of shameful memories contributing to the person's core sense of shame, thereby reducing personally felt shame as well as the fear of shame previously resulting in shame regulation strategies (123). In sum, when used in a supportive therapeutic relationship, MDMA may reduce shame through the facilitation of self-transcendent emotions, particularly compassion, and by enhancing the therapeutic bond to potentiate corrective interpersonal learning. Reductions in shame should then result in reduced experiences of social evaluative threat and reductions in the defensive, avoidant, and preparatory behaviors that are central to maintaining SAD.

## Dysfunctional Social Behavior

SAD has been associated with a variety of social behaviors that function to decrease anxiety in social situations but inadvertently induce discomfort in others and evoke desires for social distance from people with SAD (12). These interpersonal behaviors are likely to contribute to the maintenance of SAD by evoking responses in others that further reinforce the person's perceived low social status and their perceptions of defectiveness and social inadequacy [e.g., (124)]. In addition, these behaviors have been shown to interfere with the development and maintenance of the kinds of close and secure relationships that are essential to adaptive functioning (125, 126). Since people with higher levels of social anxiety have been shown to preferentially benefit from the presence of close others compared to those with lower levels of social anxiety (127), behavior that blocks the development of these close secure relationships may foster an overarching sense of social disconnection in some that further contribute to heightened arousal and reduced activation of the social engagement system. Studies informed by ethological models of social anxiety (79) frame social anxiety as a mechanism that evolved to de-escalate competition between individuals in part *via* safety behaviors such as submissive gestures like gaze aversion, slouched posture, and constricted vocal tone. Consistently, one study (128) showed that socially anxious male participants demonstrated a higher number of submissive behaviors, specifically higher vocal tone and collapsed posture, than their less anxious male counterparts while engaged in a semi-structured role-play designed to elicit competitive threat. These submissive behaviors were positively correlated with both state shame and social anxiety. In addition, Zimmerman et al. (129) found that submissive behaviors mediated the relation between trait social anxiety and internalized shame among socially anxious men. These submissive displays are inconsistent with desired behavioral norms in Western society that have historically prioritized dominance and status as the primary markers of success, particularly for men. Although behaviors such as polite smiles, directing gaze downward, restricting speech in conversation, and speaking softly may serve to reduce anxiety in the moment, they have been associated with negative perceptions from observers and decreased willingness among peers to re-engage the individual in conversation in a lab setting, particularly in ambiguous social tasks (130–132). Perhaps the most extreme behavior in the repertoire of individuals with SAD to down-regulate anxiety is to avoid social situations altogether (133), resulting in fewer opportunities to build relationships with others and inadvertently confirming perceptions of one's lack of social value. Thus, these interpersonal deficits may both impair the development of new relationships and interfere with the development of greater intimacy and safety in existing relationships, thus perpetuating perceptions of social threat due to lack of a close and connected relationships. Furthermore, research suggests that SAD severity may be associated with reduced prosocial interpersonal signals. One example is the reduced frequency of genuine smiles in social interactions. Genuine smiles have been robustly demonstrated to be perceived as signals of generosity (134), competence (135), and intrinsic engagement and motivation by others (136). In one study, people with SAD showed less smiling during social encounters compared to controls and this predicted less interest in future interactions from conversational partners (125). This lack of smiling in people with SAD is likely to result in lower liking and pleasure in conversational partners, which has been confirmed in a study that found that conversational partners low in social anxiety did not experience boosts in positive affect when paired with individuals high in social anxiety and that this lack of positive affect was correlated with SAD partner's lack of smiling (137). The heightened sense of social threat in people with SAD also leads to a tendency to conceal their true feelings, a phenomenon termed expressive suppression (138). The behavior of masking one's true feelings has been demonstrated to lead to negative reactions from others in social encounters (139–141). Furthermore, trait tendencies toward expressive suppression have been shown to predict negative interpersonal outcomes over time (126, 142) including reduced social support (143) and difficulty forming new relationships (126). Fortunately, expressive suppression has been shown to decrease with successful treatment of SAD (138), suggesting that improvements in expressive suppression may play an important role in recovery from SAD. These interpersonal deficits in SAD are also likely to lead to subjective feelings of inauthenticity and perceptions from others that the person is being inauthentic. Authenticity refers to whether a person is expressing their “true” or “core” self in their behavior or whether the person is behaving in a way that is true to what they really experience (144). Authenticity has been associated with higher personal (145–147) and interpersonal well-being (148, 149). People with SAD have been shown to experience lower self-rated authenticity in dyadic interactions as well as being rated by conversational partners as less authentic (150). Furthermore, people with SAD have also been shown to exhibit more inauthentic opinions (151), with this tendency predicting lowered self-concept clarity. This suggests that people with SAD who chronically express opinions and attitudes based on what they believe others wish to hear, rather than their own genuine viewpoint, tend to become confused about their own opinions and attitudes, potentially contributing to this uncertain and inadequate sense of self found in SAD. An individual's early social context is theorized to play a significant role in the development of safety behaviors in SAD by informing the person's sense of social-evaluative threat and level of anxiety across social situations (12). For instance, parenting styles associated with overcontrol or overprotection (152–154), exposure to adverse social experiences with peers, such as bullying, social ostracism, or peer victimization (152, 155, 156), early relational traumatic experiences (154, 157, 158) and strong societal expectations to adhere to strict cultural norms (159) contribute to the development of social anxiety and may also play a maintaining role (12). Other evidence suggests a bidirectional relation between social anxiety and social victimization and rejection, potentially due to poorer social performance secondary to increased use of safety behaviors (106, 160, 161). This may particularly be the case for children that display behaviors associated with inhibited temperament (e.g., excessive crying, shyness around strangers). Research has shown that inhibited or avoidant temperament in as early as infancy can elicit protective parental behaviors and negative interactions with same-aged peers (162, 163), that may increase the individual's threat perception of social interactions and exacerbate social-evaluative anxiety over time. A number of studies suggest that changes in safety behaviors and other forms of interpersonal expression may play an important role in recovery from SAD. For example, in one study, people with SAD engaged in an interaction with an unknown individual. They were randomly instructed to either interact normally or to reduce safety behaviors in a way intended to increase authenticity. Results showed that increases in authenticity due to reduced safety behaviors led to increased positive affect in the person with SAD, more positive perceptions of their partner's response, and a greater desire to interact with their partner again (42). Another experimental study showed that people with SAD assigned to a safety behavior reduction intervention prior to a conversation showed increases in both perceived and actual positive interpersonal outcomes, reduced self-judgments, and more social approach behavior compared to the control condition (164). Together, these results show that both authenticity and positivity can be increased in social interactions among people with SAD and that increases in authenticity have important personal and interpersonal benefits. This also suggests that it may be important for people with SAD to expose their true selves to social scrutiny and that authenticity may be an important treatment target for people with SAD. In sum, the social context of people with SAD appears to play an important role in maintaining the disorder. In addition, it appears that people with SAD contribute to their negative social context by engaging in various problematic social behaviors including reduced smiling, expressive suppression, and inauthenticity that may result in negative reactions from others and thereby confirm perceptions of low social status. Furthermore, research suggests that the behavior of close others may affect the outcome of CBT for SAD (165). Thus, interventions that help increase feelings of social safeness, increase positivity in social interactions, and reduce social threat may improve outcomes for SAD *via* changes in spontaneous interpersonal behavior and improved responses from others signaling social inclusion and liking. Increases in social reinforcement might result in more positive emotion expressed during social situations, such as genuine smiling. Increased safety and reduced shame due to MDMA-AT would presumably result in a decrease in safety behaviors or expression suppression that have been shown to impede relationship development and intimacy. Furthermore, a qualitative study of people in MDMA-AT for PTSD, Barone et al. (110) found that 12 out of 19 of the interviewees reported subjective improvements in relationships and social skills as a result of MDMA-AT. In addition, research shows that people taking MDMA feel more authentic (166) and experience enhanced positive emotions (27) that might lead to shifts in interpersonal behavior that alter their social context, resulting in a beneficial feedback loop.

## Discussion

Social anxiety disorder is a debilitating psychological problem with heavy costs to the individual and society at large. Although effective behavioral and pharmacological interventions exist, many people struggling with SAD experience ongoing symptoms following treatment (16, 167) or do not present for treatment (5) indicating room for innovation in SAD treatment strategies. We believe MDMA-AT is particularly well-situated to be a novel, efficient, and effective intervention to help individuals with SAD alleviate psychological suffering and improve social functioning. In this paper, we put forth four domains where MDMA-AT may have lasting effects in altering processes hypothesized to maintain SAD beyond the dosing session: social anhedonia and decreased social reward, heightened social threat perception, increased shame and self-criticism, and dysfunctional interpersonal behavior. Evidence considered in this review for the role of MDMA in facilitating these changes was largely from basic scientific research using non-human animals and studies assessing humans who ingested MDMA outside the context of psychotherapy. Future clinical research examining the effectiveness of MDMA-AT will be necessary to test the hypothesized effectiveness of MDMA-AT for SAD beyond the single study conducted to date (52), as well as to directly test the respective roles of enhanced social reward sensitivity, decreased social threat perception, reduced shame and self-criticism, and alterations in interpersonal behavior in facilitating MDMA-AT treatment outcomes. It is our hope that by understanding the relevant processes of change in MDMA-AT for SAD, therapists may be better equipped to tailor in-session interventions to fit the individual, thereby augmenting treatment outcomes and facilitating greater and more lasting changes in social functioning and quality of life.

## Author Contributions

JL and ML contributed to all aspects of the manuscript from initial conceptualization, drafting, and editing the manuscript. Both authors agree to be accountable for the work.

## Funding

This paper was funded by internal funding from Portland Psychotherapy Clinic, Research, and Training Clinic as part of it's social enterprise model to support scientific research.

## Conflict of Interest

The authors declare that the research was conducted in the absence of any commercial or financial relationships that could be construed as a potential conflict of interest.

## Publisher's Note

All claims expressed in this article are solely those of the authors and do not necessarily represent those of their affiliated organizations, or those of the publisher, the editors and the reviewers. Any product that may be evaluated in this article, or claim that may be made by its manufacturer, is not guaranteed or endorsed by the publisher.

## Abbreviations

MDMA-AT: MDMA-Assisted Therapy
SA: Social Anxiety
SAD: Social Anxiety Disorder.

## References

[1] Kessler RC Berglund P Demler O Jin R Merikangas KR Walters EE. Lifetime prevalence of age-of-onset distributions of DSM-IV disorders in the national comorbidity survey replication. Arch Gen Psychiatry. (2005) 62:593–602. 10.1001/archpsyc.62.6.593 15939837

[2] American Psychiatric Association (APA). Diagnostic and Statistical Manual of Mental Disorders: DSM-5. Washington, DC: American Psychiatric Association (2013).

[3] Fehm L Pelissolo A Furmark T Wittchen HU. Size and burden of social phobia in Europe. Eur Neuropsychopharmacol. (2005) 15:453–62. 10.1016/j.euroneuro.2005.04.002 15921898

[4] Fehm L Beesdo K Jacobi F Fiedler A. Social anxiety disorder above and below the diagnostic threshold: prevalence, comorbidity, and impairment in the general population. Soc Psychiatry Psychiatr Epidemiol. (2008) 43:257–65. 10.1007/s00127-007-0299-4 18084686

[5] Issakidis C Sanderson K Corry J Andrews G Lapsley H. Modelling the population cost-effectiveness of current and evidence-based optimal treatment for anxiety disorders. Psychol Med. (2004) 34:19–35. 10.1017/s003329170300881x 14971624

[6] Stuhldreher N Leibing E Leichsenring F Beutel ME Herpertz S Hoyer J. The costs of social anxiety disorder: the role of symptom severity and comorbidities. J Affect Disord. (2014) 165:87–94. 10.1016/j.jad.2014.04.039 24882183

[7] Buckner JD Schmidt NB Lang AR Small JW Schlauch RC Lewinsohn PM. Specificity of social anxiety disorder as a risk factor for alcohol and cannabis dependence. J Psychiatr Res. (2008) 42:230–9. 10.1016/j.jpsychires.2007.01.002 17320907 PMC2254175

[8] Oliveira LM Bermudez MB de Amorim Macedo MJ Passos IC. comorbid social anxiety disorder in patients with alcohol use disorder: a systematic review. J Psychiatr Res. (2018) 106:8–14. 10.1016/j.jpsychires.2018.09.008 30236640

[9] Adams GC Balbuena L Meng XF Asmundson GJG. When social anxiety and depression go together: a population study of comorbidity and associated consequences. J Affect Disord. (2016) 206:48–54. 10.1016/j.jad.2016.07.031 27466742

[10] Ohayon MM Schatzberg AF. Social phobia and depression: prevalence and comorbidity. J Psychosom Res. (2010) 3:235–43. 10.1016/j.jpsychores.2009.07.018 20159208

[11] Leigh-Hunt N Bagguley D Bash K Turner V Turnbull S Valtorta N. An overview of systematic reviews on the public health consequences of social isolation and loneliness. Public Health. (2017) 152:157–71. 10.1016/j.puhe.2017.07.035 28915435

[12] Wong QJJ Rapee RM. (2016). The aetiology and maintenance of social anxiety disorder: a synthesis of complimentary theoretical models and formulation of a new integrated model. J Affect Disord. (2016) 203:84–100. 10.1016/j.jad.2016.05.069 27280967

[13] Weiller E Bisserbe JC Boyer P Lepine JP Lecrubier Y. Social phobia in general health care: an unrecognised undertreated disabling disorder. Br J Psychiatry. (1996) 168:169–74. 10.1192/bjp.168.2.169 8837906

[14] Williams T McCaul M Schwarzer G Cipriani A Stein DJ Ipser J. Pharmacological treatments for social anxiety disorder in adults: a systematic review and network meta-analysis. Acta Neuropsychiatr. (2020) 32:169–76. 10.1017/neu.2020.6 32039743

[15] Mayo-Wilson E Dias S Mavranezouli I Kew K Clark DM Ades AE. Psychological and pharmacological interventions for social anxiety disorder in adults: a systematic review and network meta-analysis. Lancet Psychiatry. (2014) 1:368–76. 10.1016/S2215-0366(14)70329-3 26361000 PMC4287862

[16] Steinert C Hofmann M Leichsenring F Kruse J. What do we know today about the prospective long-term course of social anxiety disorder? A systematic literature review. J Anxiety Disord. (2013) 27:692–702. 10.1016/j.janxdis.2013.08.002 24176803

[17] Mataix-Cols D De La Cruz LF Monzani B Rosenfield D Andersson E Pérez-Vigil A. D-cycloserine augmentation of exposure-based cognitive behavior therapy for anxiety, obsessive-compulsive, and posttraumatic stress disorders a systematic review and meta-analysis of individual participant data. JAMA Psychiatry. (2017) 74:501–10. 10.1001/jamapsychiatry.2016.3955 28122091

[18] Bejerot S Eriksson JM. Mortberg, E. Social anxiety in adult autism spectrum disorder. Psychiatry Res. (2014) 220:705–7. 10.1016/j.psychres.2014.08.030 25200187

[19] Mitchell JM Bogenschutz M Lilienstein A Harrison C Kleiman S Parker-Guilbert K. Phase 3 MDMA-assisted therapy for severe PTSD: a randomized, double-blind, placebo-controlled phase 3 study. Nat Med. (2021) 27:1025–33. 10.1038/s41591-021-01336-3 33972795 PMC8205851

[20] Mithoefer MC Feduccia AA Jerome L Mithoefer A Wagner M Walsh Z. MDMA-assisted psychotherapy for treatment of PTSD: study design and rationale for phase 3 trials based on pooled analysis of six phase 2 randomized controlled trials. Psychopharmacology. (2019) 236:2735–45. 10.1007/s00213-019-05249-5 31065731 PMC6695343

[21] Bahji A Forsyth A Groll D Hawken ER. Efficacy of 3,4 Methylenedioxymethamphetamine (MDMA)-assisted psychotherapy for posttraumatic stress disorder: a systematic review and meta-analysis. Prog Neuropsychopharmacol Biol Psychiatry. (2020) 96:109735. 10.1016/j.pnpbp.2019.109735 31437480

[22] Heifets BD Malenka RC. MDMA as a probe and treatment for social behaviors. Cell. (2016) 166:269–72. 10.1016/j.cell.2016.06.045 27419864

[23] Passie T Hartmann U Schneider U Emrich HM Krüger THC. Ecstasy (MDMA) mimics the post-orgasmic state: impairment of sexual drive and function during acute MDMA-effects may be due to increased prolactin secretion. Med Hypotheses. (2005) 64:899–903. 10.1016/j.mehy.2004.11.044 15780482

[24] Mas M Farre M. de la Torre R Roset PN Ortuno J Segura J. Cardiovascular and neuroendocrine effects and pharmacokinetics of 3, 4-methylenedioxymethamphetamine in humans. J Pharmacol Exp Ther. (1999) 290:136–45. 10381769

[25] Emanuele E Arra M Pesenti S. Vasopressin and oxytocin as neurohormonal mediators of mdma (ecstasy) sociosexual behavioural effects. Med Hypotheses. (2006) 67:1250–1. 10.1016/j.mehy.2006.05.021 16814476

[26] Liechti ME Vollenweider FX. Which neuroreceptors mediate the subjective effects of MDMA in humans? A summary of mechanistic studies. Hum Psychopharmacol. (2001) 16:589–98. 10.1002/hup.348 12404538

[27] Studerus E Gamma A Vollenweider FX. Psychometric evaluation of the altered states of consciousness rating scale (OAV). PLoS ONE. (2010) 5:e0012412. 10.1371/journal.pone.0012412 20824211 PMC2930851

[28] Passie T. The early use of MDMA (‘Ecstasy') in Psychotherapy (1977–1985). Drug Sci Policy Law. (2018) 4:205032451876744. 10.1177/2050324518767442

[29] Hayes SC Hofmann SG Stanton CE Carpenter JK Sanford BT Curtiss JE. The role of the individual in the coming era of process-based therapy. Behav Res Ther. (2019) 117:40–53. 10.1016/j.brat.2018.10.005 30348451

[30] Gilboa-Schechtman E Shachar I Sahar Y. Positivity impairment as a broad-based feature of social anxiety. In: Weeks JW editor. The Wiley Blackwell Handbook of Social Anxiety Disorder. Hoboken, NJ: Wiley-Blackwell (2014). p. 409–32.

[31] Richey JA Brewer JA Sullivan-Toole H Strege MV Kim-Spoon J White SW. Sensitivity shift theory: a developmental model of positive affect and motivational deficits in social anxiety disorder. Clin Psychol Rev. (2019) 72:101756. 10.1016/j.cpr.2019.101756 31351312

[32] Amir N Prouvost C Kuckertz JM. (2012). Lack of a benign interpretation bias in social anxiety disorder. Cogn Behav Ther. (2012) 41:119–29. 10.1080/16506073.2012.662655 22545788 PMC3708600

[33] Caouette JD Guyer AE. Cognitive distortions mediate depression and affective response to social acceptance and rejection. J Affect Disord. (2016) 190:792–9. 10.1016/j.jad.2015.11.015 26625091 PMC4745658

[34] Gable SL Gosnell CL. (2013). Approach and avoidance behavior in interpersonal relationships. Emot Rev. (2013) 5:269–74. 10.1177/1754073913477513

[35] Gilboa-Schechtman E Keshet H Peschard V Azoulay R. (2020). Self and identity in social anxiety disorder. J Pers. (2020) 88:106–21. 10.1111/jopy.12455 30663766

[36] Beltzer ML Adams S Beling PA Teachman BA. Social anxiety and dynamic social reinforcement learning in a volatile environment. Clin Psychol Sci. (2019) 7:1372–88. 10.1177/2167702619858425 32864197 PMC7451209

[37] Kashdan TB Weeks JW Savostyanova AA. Whether, how, and when social anxiety shapes positive experiences and events: a self-regulatory framework and treatment implications. Clin Psychol Rev. (2011) 31:786–99. 10.1016/j.cpr.2011.03.012 21529701

[38] Alden LE Wallace ST. (1995). Social phobia and social appraisal in successful and unsuccessful social interactions. Behav Res Ther. (1995) 33:497–505. 10.1016/0005-7967(94)00088-2 7598670

[39] Wallace ST Alden LE. Social phobia and positive social events: the price of success. J Abnorm Psychol. (1997) 106:416. 10.1037//0021-843x.106.3.416 9241943

[40] Alden LE Taylor CT Mellings TMJB Laposa JM. Social anxiety and the interpretation of positive social events. J Anxiety Disord. (2008) 22:577–90. 10.1016/j.janxdis.2007.05.007 17587542

[41] Morrison AS Mateen MA Brozovich FA Zaki J Goldin PR Heimberg RG. Changes in empathy mediate the effects of cognitive-behavioral group therapy but not mindfulness-based stress reduction for social anxiety disorder. Behav Ther. (2019) 50:1098–111. 10.1016/j.beth.2019.05.005 31735245

[42] Plasencia ML Taylor CT Alden LE. unmasking one's true self facilitates positive relational outcomes: authenticity promotes social approach processes in social anxiety disorder. Clin Psychol Sci. (2016) 4:1002–14. 10.1177/2167702615622204

[43] Taylor CT Alden LE. To see ourselves as others see us: an experimental integration of the intra and interpersonal consequences of self-protection in social anxiety disorder. J Abnorm Psychol. (2011) 120:129. 10.1037/a0022127 21319927

[44] Jungaberle H Thal S Zeuch A Rougemont-Bücking A von Heyden M Aicher H. Positive psychology in the investigation of psychedelics and entactogens: a critical review. Neuropharmacology. (2018) 142:179–99. 10.1016/j.neuropharm.2018.06.034 29964094

[45] Carter CS Grippo AJ Pournajafi-Nazarloo H Ruscio MG Porges SW. Oxytocin, vasopressin and sociality. Prog Brain Res. (2008) 170:331–6. 10.1016/S0079-6123(08)00427-5 18655893

[46] Kemp AH Quintana DS Kuhnert R-L Griffiths K Hickie IB Guastella AJ. Oxytocin increases heart rate variability in humans at rest: implications for social approach-related motivation and capacity for social engagement. PLoS ONE. (2012) 7:e44014. 10.1371/journal.pone.0044014 22937145 PMC3429409

[47] Ago Y Nakamura S Baba A Matsuda T. Neuropsychotoxicity of abused drugs: effects of serotonin receptor ligands on methamphetamine- and cocaine-induced behavioral sensitization in mice. J Pharmacol Sci. (2008) 106:15–211. 10.1254/jphs.FM0070121 18198473

[48] Auclair A Drouin C Cotecchia S Glowinski J Tassin J-P. 5-HT2A and Alpha1b-adrenergic receptors entirely mediate dopamine release, locomotor response and behavioural sensitization to opiates and psychostimulants. Eur J Neurosci. (2004) 20:3073–84. 10.1111/j.1460-9568.2004.03805.x 15579162

[49] Zayara AE McIver G Valdivia PN Lominac KD McCreary AC Szumlinski KK. Blockade of nucleus accumbens 5-HT2A and 5-HT2C receptors prevents the expression of cocaine-induced behavioral and neurochemical sensitization in rats. Psychopharmacology (Berl). (2011) 213:321–35. 10.1007/s00213-010-1996-3 20814782 PMC3032203

[50] Nardou R Lewis EM Rothhaas R Xu R Yang A Boyden E. (2019). Oxytocin-dependent reopening of a social reward learning critical period with MDMA. Nature. (2019) 569:116–20. 10.1038/s41586-019-1075-9 30944474

[51] Curry DW Berro LF Belkoff AR Sulima A Rice KC Howell LL. Sensitization to the Prosocial Effects of 3,4-Methylenedioxymethamphetamine (MDMA). Neuropharmacology. (2019) 151:13–20. 10.1016/j.neuropharm.2019.03.017 30922893 PMC8364737

[52] Danforth AL Grob CS Struble C Feduccia AA Walker N Jerome L. Reduction in social anxiety after MDMA-assisted psychotherapy with autistic adults: a randomized, double-blind, placebo-controlled pilot study. Psychopharmacology (Berl). (2018) 235:3137–48. 10.1007/s00213-018-5010-9 30196397 PMC6208958

[53] Tiedens LZ Fragale AR. Power moves: complementarity in dominant and submissive nonverbal behavior. J Pers Soc Psychol. (2003) 84:558–68. 10.1037/0022-3514.84.3.558 12635916

[54] Blair K Geraci M Devido J McCaffrey D Chen G Vythilingam M. Neural response to self- and other referential praise and criticism in generalized social phobia. Arch Gen Psychiatry. (2008) 65:1176–84. 10.1001/archpsyc.65.10.1176 18838634 PMC2785901

[55] Lorberbaum JP Kose S Johnson MR Arana GW Sullivan LK Hamner MB. Neural correlates of speech anticipatory anxiety in generalized social phobia. Neuroreport. (2004) 15:2701–5. 15597038

[56] Phan KL Fitzgerald DA Nathan PJ Tancer ME. Association between amygdala hyperactivity to harsh faces and severity of social anxiety in generalized social phobia. Biol Psychiatry. (2006) 59:424–9. 10.1016/j.biopsych.2005.08.012 16256956

[57] Asher M Aderka IM. Dating with social anxiety: an empirical examination of momentary anxiety and desire for future interaction. Clin Psychol Sci. (2020) 8:99–110. 10.1177/2167702619867055

[58] Voncken MJ Dijk C Lang WG Boots LMM Roelofs J. Behavior when socially anxious individuals expect to be (Dis)liked: the role of self-disclosure and mimicry in actual likeability. J Behav Ther Exp Psychiatry. (2020) 69:101574. 10.1016/j.jbtep.2020.101574 32470686

[59] Kashdan TB Doorley J Stiksma MC Hertenstein MJ. Discomfort and avoidance of touch: new insights on the emotional deficits of social anxiety. Cogn Emot. (2017) 31:1638–46. 10.1080/02699931.2016.1256867 27873536

[60] Fetzner MG Teale Sapach MJN Mulvogue M Carleton RN. “It's not just about being judged”: interpersonal distrust uniquely contributes to social anxiety. J Exp Psychopathol. (2016) 7:31–40. 10.5127/jep.042414

[61] Hattingh CJ Ipser J Tromp SA Syal S Lochner C Brooks SJ. Functional magnetic resonance imaging during emotion recognition in social anxiety disorder: an activation likelihood meta-analysis. Front Hum Neurosci. (2013) 6:347. 10.3389/fnhum.2012.00347 23335892 PMC3547329

[62] Suveg C Kingery JN Davis M Jones A Whitehead M Jacob ML. Still lonely: social adjustment of youth with and without social anxiety disorder following cognitive behavioral therapy. J Anxiety Disord. (2017) 52:72–8. 10.1016/j.janxdis.2017.10.005 29069628

[63] Porges SW. The Polyvagal Theory: Neurophysiological Foundations of Emotions, Attachment, Communication, and Self-Regulation (Norton Series on Interpersonal Neurobiology). New York, NY: W.W. Norton & Company (2011). p. 347.

[64] Lynch TR. Radically Open Dialectical Behavior Therapy: Theory and Practice for Treating Disorders of Overcontrol. Oakland, CA: New Harbinger (2018). p. 520.

[65] Thayer JF Lane RDA. Model of neurovisceral integration in emotion regulation and dysregulation. J Affect Disord. (2000) 61:201–16. 10.1016/s0165-0327(00)00338-4 11163422

[66] Pittig A Arch JJ Lam CWR Craske MG. Heart rate and heart rate variability in panic, social anxiety, obsessive-compulsive, and generalized anxiety disorders at baseline and in response to relaxation and hyperventilation. Int J Psychophysiol. (2013) 87:19–27. 10.1016/j.ijpsycho.2012.10.012 23107994

[67] Alvares GA Quintana DS Kemp AH Van Zwieten A Balleine BW Hickie IB. Reduced heart rate variability in social anxiety disorder: associations with gender and symptom severity. PLoS ONE. (2013) 8e70468. 10.1371/journal.pone.0070468 23936207 PMC3728204

[68] Miles LK Nind LK Henderson Z Macrae CN. Moving memories: behavioral synchrony and memory for self and others. J Exp Soc Psychol. (2010) 46:457–60. 10.1016/j.jesp.2009.12.006

[69] Asher M Kauffmann A Aderka IM. Out of sync: nonverbal synchrony in social anxiety disorder. Clinc Psychol Sci. (2020) 8:280–94. 10.1177/2167702619894566

[70] Anderl C Steil R Hahn T Hitzeroth P Reif A Windmann S. Reduced reciprocal giving in social anxiety - evidence from the trust game. J Behav Ther Exp Psychiatry. (2018) 59:12–8. 10.1016/j.jbtep.2017.10.005 29121505

[71] Ketay S Welker KM Beck LA Thorson KR Slatcher RB. Social anxiety, cortisol, and early-stage friendship. J Soc Pers Relat. (2019) 36:1954–74. 10.1177/0265407518774915

[72] Alden LE Buhr K Robichaud M Trew JL Plasencia ML. Treatment of social approach processes in adults with social anxiety disorder. J Consult Clin Psychol. (2018) 86:505–17. 10.1037/ccp0000306 29781649

[73] Shahar B Bar-Kalifa E Alon E. Emotion-focused therapy for social anxiety disorder: results from a multiple-baseline study. J Consult Clin Psychol. (2017) 85:238–49. 10.1037/ccp0000166 28221059

[74] Trew JL Alden LE. Kindness reduces avoidance goals in socially anxious individuals. Motiv Emot. (2015) 39:892–907. 10.1007/s11031-015-9499-5

[75] Vernberg EM Abwender DA Ewell KK Beery SH. Social anxiety and peer relationships in early adolescence: a prospective analysis. J Clin Child Psychol. (1992) 21:189–96. 10.1207/s15374424jccp2102_11

[76] Morrison AS Mateen MA Brozovich FA Zaki J Goldin PR Heimberg RG. Empathy for positive and negative emotions in social anxiety disorder. Behav Res Ther. (2016) 87:232–42. 10.1016/j.brat.2016.10.005 27816799 PMC5142818

[77] Arditte Hall KA Joormann J Siemer M Timpano KR. The impact bias in self and others: affective and emptathic forecasting in individuals with social anxiety. Behav Res Ther. (2018) 106:37–46. 10.1016/j.brat.2018.05.001 29758391

[78] Barone WJ. The role of MDMA as an adjunct to therapy for adults with PTSD, as illustrated by participant qualitative data one-year posttreatment (dissertation). Pleasant Hill, CA: John F. Kennedy University (2017).

[79] Gilbert P. The relationship of shame, social anxiety and depression: the role of the evaluation of social rank. Clin Psychol Psychother. (2000) 7:174–89. 10.1002/1099-0879(200007)7:3<174::AID-CPP236>3.0.CO;2-U

[80] Weeks JW Howell AN Srivastav A Goldin PR. “Fear guides the eyes of the beholder”: assessing gaze avoidance in social anxiety disorder *via* covert eye tracking of dynamic social stimuli. J Anxiety Disord. (2019) 65:56–63. 10.1016/j.janxdis.2019.05.005 31170596

[81] Reichenberger J Wiggert N Wilhelm FH Liedlgruber M Voderholzer U Hillert A. Fear of negative and positive evaluation and reactivity to social-evaluative videos in social anxiety disorder. Behav Res Ther. (2019) 116:140–8. 10.1016/j.brat.2019.03.009 30921745

[82] Bershad AK Miller MA Baggott MJ De Wit H. The effects of MDMA on socio-emotional processing: does MDMA differ from other stimulants? J Psychopharmacol. (2016) 30:1248–58. 10.1177/0269881116663120 27562198 PMC8753974

[83] Kok BE Fredrickson BL. Upward spirals of the heart: autonomic flexibility, as indexed by vagal tone, reciprocally and prospectively predicts positive emotions and social connectedness. Biol Psychol. (2010) 85:432–6. 10.1016/j.biopsycho.2010.09.005 20851735 PMC3122270

[84] Arai A Hirota Y Miyase N Miyata S Young LJ Osako Y. A single prolonged stress paradigm produces enduring impairments in social bonding in monogamous prairie voles. Behav Brain Res. (2016) 315:83–93. 10.1016/j.bbr.2016.08.022 27522019 PMC5226121

[85] Tangney JP Dearing RL. Shame and Guilt. New York, NY: The Guilford Press (2002). p. 272.

[86] Cândea DM Szentágotai-Tǎtar A. The impact of self-compassion on shame-proneness in social anxiety. Mindfulness. (2018) 9:1816–24. 10.1007/s12671-018-0924-1

[87] Shahar B Doron G Szepsenwol O. Childhood maltreatment, shame-proneness and self-criticism in social anxiety disorder: a sequential mediational model. Clin Psychol Psychother. (2015) 22:570–9. 10.1002/cpp.1918 25196782

[88] Fergus TA Valentiner DP McGrath PB Jencius S. Shame- and guilt-proneness: relationships with anxiety disorder symptoms in a clinical sample. J Anxiety Disord. (2010) 24:811–5. 10.1016/j.janxdis.2010.06.002 20591613

[89] Gilbert P. The evolution of social attractiveness and its role in shame, humiliation, guilt and therapy. Br J Med Psychol. (1997) 70:113–47. 10.1111/j.2044-8341.1997.tb01893.x 9210990

[90] Sznycer D Tooby J Cosmides L Porat R Shalvi S Halperin E. Shame closely tracks the threat of devaluation by others, even across cultures. Proc Natl Acad Sci. (2016) 113:2625–30. 10.1073/pnas.1514699113 26903649 PMC4790975

[91] Robertson TE Sznycer D Delton AW Tooby J Cosmides L. The true trigger of shame: social devaluation is sufficient, wrongdoing is unnecessary. Evol Hum Behav. (2018) 39:566–73. 10.1016/j.evolhumbehav.2018.05.010

[92] Hedman E Ström P Stünkel A Mörtberg E. Shame and guilt in social anxiety disorder: effects of cognitive behavior therapy and association with social anxiety and depressive symptoms. PLoS ONE. (2013) 8:e61713. 10.1371/journal.pone.0061713 23620782 PMC3631156

[93] Haberman A Shahar B Bar-Kalifa E Zilcha-Mano S Diamond GM. Exploring the process of change in emotion-focused therapy for social anxiety. Psychother Res. (2019) 29:908–18. 10.1080/10503307.2018 29366385

[94] Moscovitch DA. (2009). What is the core fear in social phobia? A new model to facilitate individualized case conceptualization and treatment. Cogn Behav Pract. (2009) 16:123–34. 10.1016/j.cbpra.2008.04.002

[95] Lazarus G Shahar B. (2018). The role of shame and self-criticism in social anxiety: a daily diary study in a nonclinical sample. J Soc Clin Psychol. (2018) 37:107–27. 10.1521/jscp.2018.37.2.107

[96] Cândea DM Szentágotai-Tătar A. Shame as a predictor of post-event rumination in social anxiety. Cogn Emot. (2017) 31:1684–91. 10.1080/02699931.2016.1243518 27744788

[97] Shahar B. Emotion focused therapy for the treatment of social anxiety: an overview of the model and a case description. Clin Psychol Psychother. (2014) 21:536–47. 10.1002/cpp.1853 23813629

[98] Brozovich F Heimberg RG. An analysis of postevent processing in social anxiety disorder. Clin Psychol Rev. (2008) 28:891–903. 10.1016/j.cpr.2008.01.002 18294745

[99] Longe O Maratos FA Gilbert P Evans G Volker F Rockliff H. Having a word with yourself: neural correlates of self-criticism and self-reassurance. Neuroimage. (2010) 49:1849–56. 10.1016/i.neuroimage.2009.09.019 19770047

[100] Anderson B Goldin PR Kurita K Gross JJ. Self-representation in social anxiety disorder: linguistic analysis of autobiographical narratives. Behav Res Ther. (2008) 46:1119–25. 10.1016/j.brat.2008.07.00 18722589 PMC2630512

[101] Makkar SR Grisham JR. Social anxiety and the effects of negative self-imagery on emotion, cognition, and post-event processing. Behav Res Ther. (2011) 49:654–64. 10.1016/j.brat.2011.07.004 21788011

[102] Stopa L Jenkins A. Images of the self in social anxiety: effects on the retrieval of autobiographical memories. J Behav Ther Exp Psychiatry. (2007) 38:459–73. 10.1016/j.jbtep.2007.08.006 17931596

[103] Matos M Pinto-Gouveia J Gilbert P. The effect of shame and shame memories on paranoid ideation and social anxiety. Clin Psychol Psychother. (2013) 20:334–49. 10.1002/cpp.1766 22290772

[104] Zoccola PM Dickerson SS Lam S. Eliciting and maintaining ruminative thought: the role of social-evaluative threat. Emotion. (2012) 12:673–7. 10.1037/a0027349 22390710

[105] Hofmann SG. Self-focused attention before and after treatment of social phobia. Behav Res Ther. (2000) 38:717–25. 10.1016/S0005-7967(99)00105-9 10875193

[106] Norton AR Abbott MJ. Self-focused cognition in social anxiety: a review of the theoretical and empirical literature. Behav Change. (2015) 33:44–64. 10.1017/bec.2016.2

[107] Gregory B Peters L. Changes in the self during cognitive behavioural therapy for social anxiety disorder: a systematic review. Clin Psychol Rev. (2017) 52:1–18. 10.1016/j.cpr.2016.11.008 27912159

[108] Kamboj SK Kilford EJ Minchin S Moss A Lawn W Das RK. Recreational 3,4- methylenedioxy-N-methylamphetamine (MDMA) or ecstasy and self-focused compassion: preliminary steps in the development of a therapeutic psychopharmacology of contemplative practices. J Psychopharmacol. (2015) 29:961–70. 10.1177/0269881115587143 25990558

[109] Kamboj SK Walldén YSE Falconer CJ Alotaibi MR Blagbrough IS Husbands SM. Additive effects of 3,4-methylenedioxymethamphetamine (MDMA) and compassionate imagery on self-compassion in recreational users of ecstasy. Mindfulness. (2018) 9:1134–45. 10.1007/s12671-017-0849-0 30100932 PMC6061231

[110] Barone W Beck J Mitsunaga-Whitten M Perl P. Perceived benefits of MDMA-assisted psychotherapy beyond symptom reduction: qualitative follow-up study of a clinical trial for individuals with treatment-resistant PTSD. J Psychoactive Drugs. (2019) 51:199–208. 10.1080/02791072.2019.1580805 30849288

[111] Werner KH Jazaieri H Goldin PR Ziv M Heimberg RG Gross JJ. Self-compassion and social anxiety disorder. Anxiety Stress Coping. (2012) 25:543–58. 10.1080/10615806.2011.608842 21895450 PMC4128472

[112] Makadi E Koszycki D. Exploring connections between self-compassion, mindfulness, and social anxiety. Mindfulness. (2020) 11:480–92. 10.1007/s12671-019-01270-z

[113] Goetz JL Keltner D Simon-Thomas E. Compassion: an evolutionary analysis and empirical review. Psychol Bull. (2010) 136:351–74. 10.1037/a0018807 20438142 PMC2864937

[114] Piff PK Dietze P Feinberg M Stancato DM Keltner D. Awe, the small self, and prosocial behavior. J Pers Soc Psychol. (2015) 108:883–99. 10.1037/pspi0000018 25984788

[115] Shiota MN Keltner D Mossman A. The nature of awe: elicitors, appraisals, and effects on self-concept. Cogn Emot. (2007) 21:944–63. 10.1080/02699930600923668

[116] Stellar JE Gordon AM Piff PK Cordaro D Anderson CL Bai Y. Self- transcendent emotions and their social functions: compassion, gratitude, and awe bind us to others through prosociality. Emot Rev. (2017) 9:200–7. 10.1177/1754073916684557

[117] Hutcherson CA Seppala EM Gross JJ. Loving-kindness meditation increases social connectedness. Emotion. (2008) 8:720–4. 10.1037/a0013237 18837623

[118] Kok BE Singer T. Effects of contemplative dyads on engagement and perceived social connectedness over 9 months of mental training a randomized clinical trial. JAMA Psychiatry. (2017) 74:126–34. 10.1001/jamapsychiatry.2016.3360 28030741

[119] Shahar B Szsepsenwol O Zilcha-Mano S Haim N Zamir O Levi-Yeshuvi S. A wait-list randomized controlled trial of loving-kindness meditation programme for self-criticism. Clin Psychol Psychother. (2014) 22:346–56. 10.1002/cpp.1893 24633992

[120] Markey PM Funder DG Ozer DJ. Complementarity of interpersonal behaviors in dyadic interactions. Pers Soc Psychol Bull. (2003) 29:1082–90. 10.1177/0146167203253474 15189605

[121] Sadler P Ethier N Gunn GR Duong D Woody E. Are we on the same wavelength? Interpersonal complementarity as shared cyclical patterns during interactions. J Pers Soc Psychol. (2009) 97:1005–20. 10.1037/a0016232 19968416

[122] Hysek CM Schmid Y Simmler LD Domes G Heinrichs M Eisenegger C. MDMA enhances emotional empathy and prosocial behavior. Soc Cogn Affect Neurosci. (2014) 9:1645–52. 10.1093/scan/nst161 24097374 PMC4221206

[123] Luoma JB Shahar B Lear MK Pilecki B Wagner A. (Under Review). Potential Processes of Change in MDMA-Assisted Psychotherapy for Social Anxiety Disorder During Acute Dosing: Enhanced Memory Reconsolidation, Self-Transcendence, and Enhanced Therapeutic Relationship. 10.1002/hup.2824 PMC9285360 34739165

[124] Voncken MJ Bögels SM. Social performance deficits in social anxiety disorder: reality during conversation and biased perception during speech. J Anxiety Disord. (2008) 22:1384–92. 10.1016/j.janxdis.2008.02.001 18343089

[125] Pearlstein SL Taylor CT Stein MB. Facial affect and interpersonal affiliation: displays of emotion during relationship formation in social anxiety disorder. Clin Psychol Sci. 7:826–39. 10.1177/2167702619825857 31565542 PMC6764757

[126] Srivastava S Tamir M McGonigal KM John OP Gross JJ. The social costs of emotional suppression: a prospective study of the transition to college. J Pers Soc Psychol. (2009) 96:883–97. 10.1037/a0014755 19309209 PMC4141473

[127] Hur J Deyoung KA Islam S Anderson AS Barstead MG Shackman AJ. Social context and the real-world consequences of social anxiety. Psychol Med. (2020) 50:1989–2000. 10.1017/S0033291719002022 31423954 PMC7028452

[128] Weeks JW Heimberg RG Heuer R. Exploring the role of behavioral submissiveness in social anxiety. J Soc Clin Psychol. (2011) 30:217–49. 10.1521/jscp.2011.30.3.217

[129] Zimmerman J Morrison AS Heimberg RG. Social anxiety, submissiveness, and shame in men and women: a moderated mediation analysis. Br J Clin Psychol. (2015) 54:1–15. 10.1111/bjc.12057 24866818

[130] Hirsch C Meynen T Clark D. Negative self-imagery in social anxiety contaminates social interactions. Memory. (2004) 12:496–506. 10.1080/09658210444000106 15487545

[131] Thompson S Rapee RM. The effect of situational structure on the social performance of socially anxious and non-anxious participants. J Behav Ther Exp Psychiatry. (2002) 33:91–102. 10.1016/s0005-7916(02)00021-6 12472173

[132] Voncken MJ Dijk KFL. Socially anxious individuals get a second chance after being disliked at first sight: the role of self-disclosure in the development of likeability in sequential social contact. Cognit Ther Res. (2013) 37:7–17. 10.1007/s10608-012-9449-4 23355754 PMC3555246

[133] Blalock DV Kashdan TB McKnight PE. High risk, high reward: daily perceptions of social challenge and performance in social anxiety disorder. J Anxiety Disord. (2018) 54:57–64. 10.1016/j.janxdis.2018.01.006 29421373

[134] Mehu M Little AC Dunbar RM. duchenne smiles and the perception of generosity and sociability in faces. J Evol Psychol. (2007) 5:183–96. 10.1556/JEP.5.2007.1-4.11

[135] Woodzicka JA. (2008). Sex differences in self-awareness of smiling during a mock job interview. J Nonverbal Behav. (2008) 32:109–21. 10.1007/s10919-007-0046-2

[136] Cheng Y Mukhopadhyay A Williams P. Smiling signals intrinsic motivation. J Consum Res. (2020) 46:915–35. 10.1093/jcrucz023

[137] Heerey EA Kring AM. Interpersonal consequences of social anxiety. J Abnorm Psychol. (2007) 116:125. 10.1037/0021-843X.116.1.125 17324023

[138] Dryman MT Heimberg RG. Emotion regulation in social anxiety and depression: a systematic review of expressive suppression and cognitive reappraisal. Clin Psychol Rev. (2018) 65:17–42. 10.1016/j.cpr.2018.07.004 30064053

[139] Butler EA Egloff B Wlhelm FH Smith NC Erickson EA Gross JJ. The social consequences of expressive suppression. Emotion. (2003) 3:48–67. 10.1037/1528-3542.3.1.48 12899316

[140] Schneider KG Hempel RJ Lynch TR. That “poker face” just might lose you the game! the impact of expressive suppression and mimicry on sensitivity to facial expressions of emotion. Emotion. (2013) 13:852–66. 10.1037/a0032847 23795586

[141] Tackman AM Srivastava S. Social responses to expressive suppression: the role of personality judgments. J Pers Soc Psychol. (2016) 110:574–91. 10.1037/pspp0000053 26280841

[142] Gross JJ John OP. Individual differences in two emotion regulation processes: implications for affect, relationships, and well-being. J Pers Soc Psychol. (2003) 85:348–62. 10.1037/0022-3514.85.2.348 12916575

[143] John OP Gross JJ. Healthy and unhealthy emotion regulation: personality processes, individual differences, and life span development. J Pers. (2004) 72:1301–33. 10.1111/j.1467-6494.2004.00298.x 15509284

[144] Ryan WS Ryan RM. Toward a social psychology of authenticity: exploring within- person variation in autonomy, congruence, and genuineness using self-determination theory. Rev Gen Psychol. (2019) 23:99–112. 10.1037/gpr0000162

[145] Lakey CE Kernis MH Heppner WL Lance CE. Individual differences in authenticity and mindfulness as predictors of verbal defensiveness. J Res Pers. (2008) 42:230–8. 10.1016/j.jrp.2007.05.002

[146] Schlegel RJ Hicks JA. The true self and psychological health: emerging evidence and future directions. Soc Personal Psychol Compass. (2011) 5:989–1003. 10.1111/j.1751-9004.2011.00401.x

[147] Wood AM Linley PA Maltby J Baliousis M Joseph S. The authentic personality: a theoretical and empirical conceptualization and the development of the authenticity scale. J Couns Psychol. (2008) 55:385–99. 10.1037/0022-0167.55.3.385

[148] Brunell AB Kernis MH Goldman BM Heppner W Davis P Cascio EV. Dispositional authenticity and romantic relationship functioning. Pers Indiv Dif. (2010) 48:900–5. 10.1016/j.paid.2010.02.018

[149] Baker ZG Tou RY Bryan JL Knee CR. Authenticity and well-being: exploring positivity and negativity in interactions as a mediator. Pers Individ Dif . (2017) 113:235–9. 10.1016/j.paid.2017.03.018

[150] Asher M Aderka IM. How real do you feel? Self- and partner-authenticity in social anxiety disorder. J Abnorm Psychol. (2021) 130:166–76. 10.1037/abn0000622 33346678

[151] Orr EMJ Moscovitch DA. Blending in at the cost of losing oneself: dishonest self- disclosure erodes self-concept clarity in social anxiety. J Exp Psychopathol. (2015) 6:278–96. 10.5127/jep.044914

[152] Rapee R Spence S. The etiology of social phobia: empirical evidence and an initial model. Clin Psychol Rev. (2004) 24:737–67. 10.1016/j.cpr.2004.06.004 15501555

[153] Kearney CA. The etiology of social anxiety and social phobia in youths. In: Kearney CA editor. Social Anxiety and Social Phobia in Youth: Characteristics, Assessment, and Psychological Treatment. New York, NY: Springer Publishing Co. (2005). p. 49–70.

[154] Kimbrel N. A model of the development and maintenance of generalized social phobia. Clin Psychol Rev. (2008) 28:592–612. 10.1016/j.cpr.2007.08.003 17959288

[155] Erwin BA Heimberg RG Marx BP Franklin ME. Traumatic and socially stressful life events among persons with social anxiety disorder. J Anxiety Disord. (2006) 20:896–914. 10.1016/j.janxdis.2005.05.006 16490343

[156] Pinto-Gouveia J Matos M. Can shame memories become a key to identity? The centrality of shame memories predicts psychopathology. Appl Cogn Psychol. (2011) 25:281–90. 10.1002/acp.1689

[157] Rosellini A Rutter L Bourgeois M Emmert-Aronson B Brown T. The relevance of age of onset to the psychopathology of social phobia. J Psychopathol Behav Assess. (2013) 35:356–65. 10.1007/s10862-013-9338-5 23935239 PMC3736863

[158] Simon N Herlands N Marks E Mancini C Letamendi A Zhonghe L. Childhood maltreatment linked to greater symptom severity and poorer quality of life and function in social anxiety disorder. Depress Anxiety. (2009) 26:1027–32. 10.1002/da.20604 19750554 PMC2991116

[159] Heinrichs N Rapee RM Alden LA Bögels SM Hofmann SG Oh KJ. Cultural differences in perceived social norms and society anxiety. Behav Res Ther. (2006) 44:1187–97. 10.1016/j.brat.2005.09.006 16325145

[160] Blöte A Miers A Westenberg P. The role of social performance and physical attractiveness in peer rejection of socially anxious adolescents. J Res Adolesc. (2015) 25:189–200. 10.1111/jora.12107

[161] Pabian S Vandebosch H. An investigation of short-term longitudinal associations between social anxiety and victimization and perpetration of traditional bullying and cyberbullying. J Youth Adolesc. (2015) 45:328–39. 10.1007/s10964-015-0259-3 25687265

[162] Kiff CJ Lengua LJ Zalewski M. Nature and nurturing: parenting in the context of child temperament. Clin Child Fam Psychol Rev. (2011) 14:251–301. 10.1007/s10567-011-0093-4 21461681 PMC3163750

[163] Rubin KH Coplan RJ Bowker JC. Social withdrawal in childhood. Annu Rev Psychol. (2009) 60:141–71. 10.1146/annurev.psych.60.110707.163642 18851686 PMC3800115

[164] Alden LE Taylor CT. Relational treatment strategies increase social approach behaviors in patients with generalized social anxiety disorder. J Anxiety Disord. (2011) 25:309–18. 10.1016/j.janxdis.2010.10.003 21094019

[165] Fogler JM Tompson MC Steketee G Hofmann SG. Influence of expressed emotion and perceived criticism on cognitive-behavioral therapy for social phobia. Behav Res Ther. (2007) 45:235–49. 10.1016/j.brat.2006.03.002 16635478

[166] Baggott MJ Coyle JR Siegrist JD Garrison KJ Galloway GP Mendelson JE. Effects of 3,4-Methylenedioxymethamphetamine on socioemotional feelings, authenticity, and autobiographical disclosure in healthy volunteers in a controlled setting. J Psychopharmacol. (2016) 30:378–87. 10.1177/0269881115626348 26880224

[167] Davidson JRT. Pharmacotherapy of social phobia. Acta Psychiatr Scand Suppl. (2003) 65–71. 10.1034/j.1600-0447.108.s417.7.x 12950437

